# Deprescribing anticholinergics to preserve brain health: reducing the risk of dementia through deprescribing (R2D2): study protocol for a randomized clinical trial

**DOI:** 10.1186/s13063-024-08618-4

**Published:** 2024-11-22

**Authors:** Noll L. Campbell, Richard J. Holden, Sujuan Gao, Frederick W. Unverzagt, Kathleen A. Lane, Allie Carter, Addison B. Harrington, Sneha Manoharan, Neha Manoharan, Danielle L. Rosenthal, Christopher Pitts, Kathryn Pelkey, Emily Papineau, David M. Lauck, Noha Keshk, Khalid Alamer, Hussein Khalil, Malaz A. Boustani

**Affiliations:** 1 Department of Pharmacy Practice, Purdue University College of Pharmacy, West Lafayette, IN USA; 2 Indiana University Center for Aging Research, Regenstrief Institute, Inc, Indianapolis, IN USA; 3 Center for Health Innovation and Implementation Science, Indiana University, Indianapolis, IN USA; 4 Department of Health & Wellness Design, Indiana University School of Public Health, Bloomington, IN USA; 5 Department of Biostatistics and Health Data Science, Indiana University School of Medicine, Indianapolis, IN USA; 6 Department of Psychiatry, Indiana University School of Medicine, Indianapolis, IN USA; 7 Department of Pharmacy, Indiana University Health, Indianapolis, IN USA; 8 Department of Pharmacy, Community Health Network, Indianapolis, IN USA; 9 Department of Medicine, Community Health Network, Indianapolis, IN USA; 10 Pharmacy Practice Department, College of Clinical Pharmacy, Imam Abdulrahman Bin Faisal University, Dammam, Saudi Arabia; 11 Department of Medicine, Indiana University School of Medicine, Indianapolis, IN USA

**Keywords:** Anticholinergics, Deprescribing, Cognition, Dementia

## Abstract

**Background:**

Older adults commonly experience chronic medical conditions and are at risk of cognitive impairment as a result of age, chronic comorbidity, and medications prescribed to manage multiple chronic conditions. Anticholinergic medications are common treatments for chronic conditions and have been repeatedly associated with poor cognitive outcomes, including delirium and dementia, in epidemiologic studies. However, no study has definitively evaluated the causal relationship between anticholinergics and cognition in a randomized controlled trial design. Utilizing our prior experience in deprescribing anticholinergic medications in various clinical environments, we designed an outpatient deprescribing intervention to prospectively test the potential causal relationship between anticholinergics and cognition in primary care older adults.

**Methods:**

This cluster randomized clinical trial will be conducted to evaluate the impact of an anticholinergic deprescribing intervention compared to usual care on outcomes of cognition and safety in primary care older adults. Participants will include those aged 65 years and over, receiving primary care in the greater Indianapolis area, using a strong anticholinergic within the last 2 weeks or with evidence of high-risk exposure in the past year. Those excluded will have a diagnosis of Alzheimer’s disease or related dementia, or serious mental illness. The trial plans to enroll 344 participants who will be cluster-randomized at the level of primary care physician to avoid contamination. Participants will complete outcome assessments every 6 months up to 2 years by blinded outcome assessors. The primary outcome of the study is a composite measure of cognition that includes domains assessing executive cognitive function, language, and memory. Secondary outcomes include patient-reported measures of pain intensity, depression, anxiety, sleep disturbance, and health-related quality of life.

**Discussion:**

The R2D2 trial will be the largest and longest prospective randomized trial testing the impact of an anticholinergic-specific deprescribing intervention on cognition in primary care older adults. Results could influence deprescribing methodology and provide new insight on the relationship between anticholinergics and cognition.

**Trial registration:**

ClinicalTrials.gov NCT04270474. Registered on February 17, 2020.

**Supplementary Information:**

The online version contains supplementary material available at 10.1186/s13063-024-08618-4.

## Background

More than 6 million older adults annually experience disability, hospitalization, or death due to a medication-related adverse event [1–4]. Up to 30% of older Americans use at least one strong anticholinergic medication every year [5, 6]. These medications are associated with increased risks of delirium, general cognitive impairment, and Alzheimer’s disease and related disorders (ADRD) [6–16], leading to higher morbidity, mortality, and healthcare costs [17].

Medications with anticholinergic activity block cholinergic receptors in the brain and have been associated with increased pathology thought to cause ADRD, with evidence from both mouse models and human studies [18–24]. Structural changes in human brain imaging studies have also been identified among users of anticholinergics compared with non-users [25]. Although a specific mechanism connecting anticholinergics with ADRD remains to be proven, several epidemiologic studies in humans show that anticholinergics increase the risk of ADRD among older adults receiving primary care [7–11, 13–16]. However, these association studies lack evidence for a causal relationship between anticholinergics and ADRD. This warrants the pursuit of prospective “deprescribing” trials to determine whether changing the potential risk factor (anticholinergic medication use) has an influence on cognition or important clinical outcomes related to brain health and patient safety.

Despite recommendations that anticholinergics should not be used as first-line treatments in older adults, no deprescribing guidelines exist to support the safe and effective reduction of these high-risk medications. Prior studies have evaluated deprescribing interventions in reducing exposure to anticholinergics [26–30]. These studies targeted a variety of populations and medications, including those with pre-existing ADRD, with some designed as feasibility studies with small sample sizes and short duration. However, two studies identified benefit in cognition in subgroups[26, 30].

Prior deprescribing trials suggest that pharmacist-based and educational interventions can safely deprescribe anticholinergics; however, the impact on clinical outcomes, namely performance on cognitive measures or the onset or diagnosis of ADRD, has not been tested in clinical trials [5, 26–28, 31, 32]. Furthermore, prior trials have not focused exclusively on strong anticholinergics, which hold the highest binding affinity to cholinergic receptors and would provide the strongest test of the link between anticholinergic medication and adverse cognitive effects [33, 34].

Therefore, we designed and initiated the randomized clinical trial, “Reducing the Risk of Dementia through Deprescribing” (R2D2) to evaluate the impact of a pharmacist-based deprescribing intervention on important cognitive and safety outcomes. The primary aim for the R2D2 trial is to determine the efficacy of anticholinergic deprescribing on objectively measured cognition compared to usual care in community-dwelling older adults. The secondary aim is to examine the quality of life and safety profile of anticholinergic deprescribing compared to usual care.

**Fig. 1:**
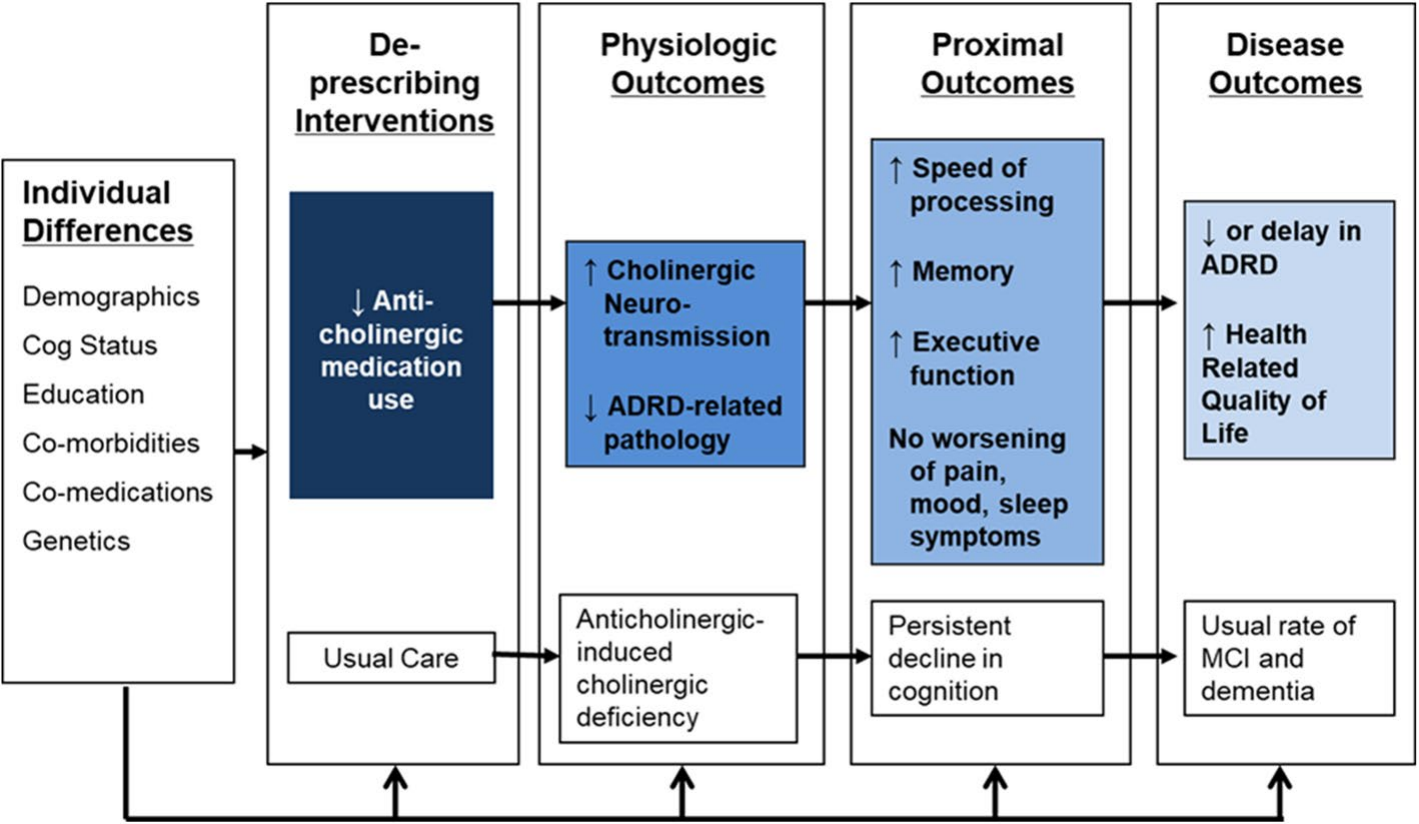
Conceptual model of the R2D2 trial

Our conceptual model (Fig. 1) holds that removal of offending anticholinergic agents will lead to increased cholinergic neurotransmission and preserved or improved cognitive function including memory, executive cognitive ability, and speed of processing. The enhancement in neural function and cognitive function produced by the intervention should result in a lower risk of cognitive impairment or delay in the time to ADRD. This trial will focus on proximal outcomes with future studies planned to evaluate the impact on disease outcomes (i.e., diagnosis of ADRD).

## Methods

The R2D2 trial's primary objective is to determine the efficacy of an anticholinergic deprescribing intervention on cognition compared to usual care in older adults using strong anticholinergics at baseline. The second objective is to examine the quality of life and safety profile of deprescribing compared to usual care. Third, the study intends to determine whether demographic (age, gender, race), genetic (APOE Ɛ4 status), or clinical characteristics (comorbidities, number of medications) moderate the effect of the deprescribing intervention.

### Study design

**Fig. 2:**
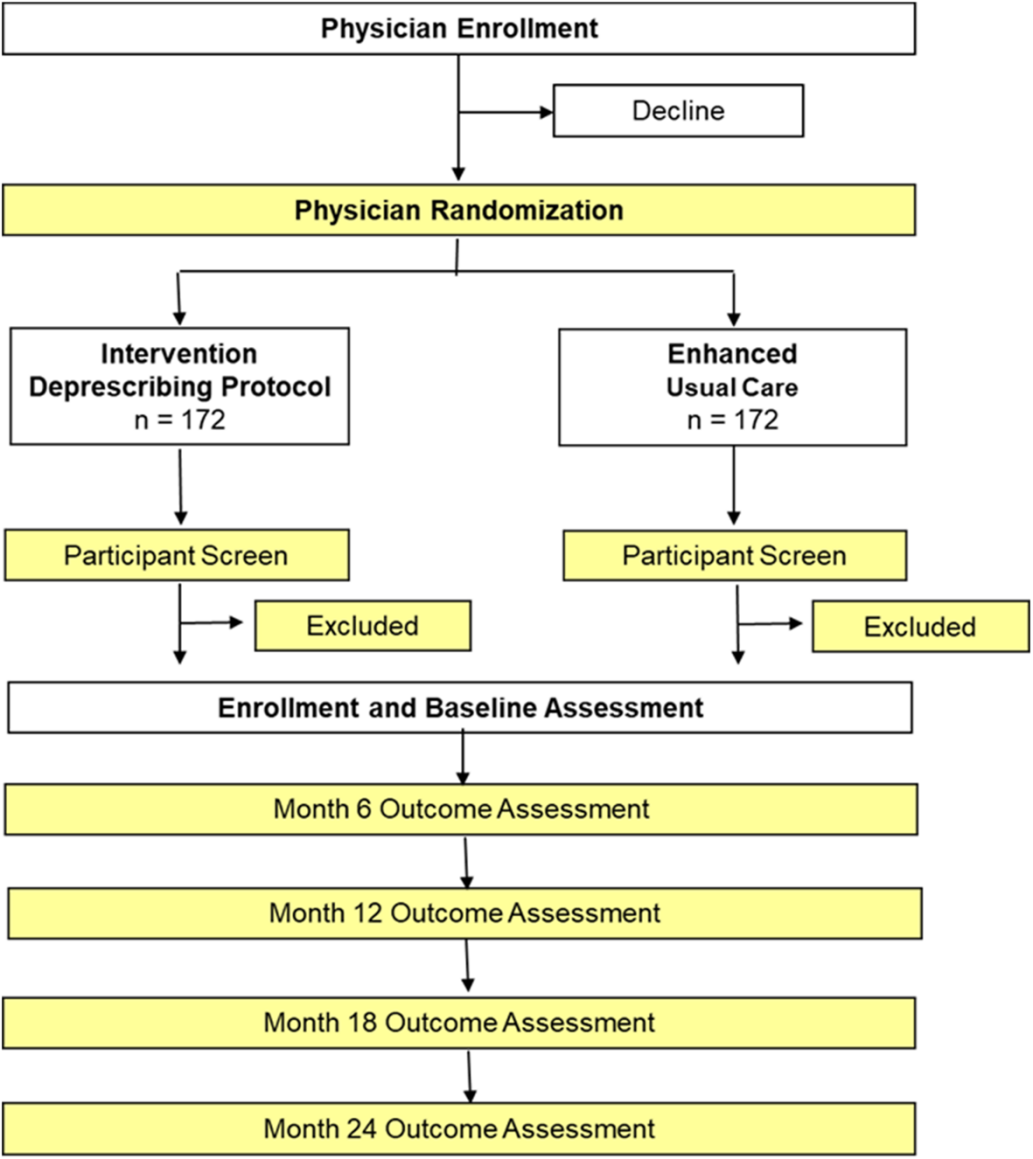
Overview of the cluster randomized design and outcome assessments

This trial adopts a cluster randomized, superiority design of a deprescribing intervention delivered over 24 months in primary care patients. The clustering component is intended to minimize the potential for contamination and is conducted at the level of primary care provider. Figure 2 provides an overview of the general study design noting the level of randomization and schedule of outcome assessments. A unique feature of the design intended to address a key gap in the existing literature is the long duration of follow-up, allowing us to detect trends in changes in outcomes up to 24 months.

### Study sites and recruitment procedures

Recruitment is conducted within primary care practices of health systems in central Indiana, including Indiana University Health and the Community Health Network. Indiana University Health is a large, quaternary care state-wide health system. Primary care locations in the greater Indianapolis area were prioritized based on the original plan for in-person recruitment. Community Health Network is a large tertiary care regional network located in central Indiana. Select primary care locations in the greater Indianapolis area were invited to participate based on potentially eligible population density and availability of support from clinical pharmacists.

**Fig. 3:**
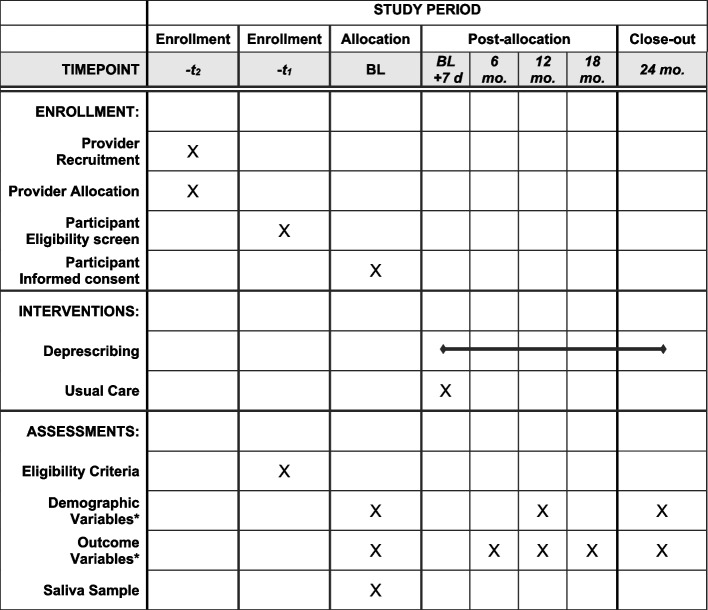
Schedule of enrollment, interventions, and assessments

Primary care leaders provide approval to conduct proposed studies in primary care environments and within patient panels. Searchable elements from electronic medical records are used to identify providers with potentially eligible patients and group those lists of patients by providers. While we do not collect data directly from providers, the intervention may influence the care some of their patients receive (particularly those providers randomized to the intervention group), making it ethically appropriate to allow providers the opportunity to opt out. Thus, prior to recruitment of potentially eligible patients, providers receive information about the study objectives, procedures, and expectations and are offered an opportunity to not participate (thus excluding all patients under their care) or exclude some of their patients. See Fig. 3 for the timeline of recruitment, intervention, and assessment of the R2D2 trial. This opt out approach was recommended by primary care leadership at participating sites.

Recruitment of potentially eligible patients utilizes lists generated from searchable criteria from electronic medical records, including age, medication and visit history, diagnostic data, and participating provider. With telephone-based recruitment, the informed consent form and HIPAA forms are provided to the participant by postal or electronic mail, or left at an agreed-upon location based on the participants’ comfort. The person obtaining consent arranges a time to discuss the study with the participant by telephone. During this telephone meeting, the person obtaining consent reviews the informed consent and HIPAA and answers any questions. After all questions have been addressed, the participant is asked to sign and date these study documents, then to return them by mail or email. The personnel obtaining consent signs and dates each returned document and proceeds with study procedures. This person is blinded to randomization status.

### Eligibility criteria

Eligible individuals are community-dwelling older adults without a diagnosis of ADRD who receive primary care from two regional healthcare systems in Indiana. We intentionally exclude those with ADRD because prior work suggests this population is not likely to benefit from the intervention [16, 26, 35].

Inclusion criteria:

1. Age 65 years and older
2. At least one office visit to their primary care physician within the previous 12 months
3. Use of at least one strong anticholinergic medication in the previous 2 weeks or a ratio of total standardized days exposed within the prior 365 days of at least 0.4 or higher [7].
4. Original criteria required the presence of subjective cognitive dysfunction by meeting either (a) or (b) of the following criteria:
  - A response of “somewhat worse” or “much worse” to the SCD screening question: “In general, how would you describe your memory as compared to 10 years ago?” Response offerings are as follows: much better/somewhat better/about the same/somewhat worse/much worse. This item is derived from the Sydney Memory and Aging study [36] and is comparable to items used to document self-report of memory in the HRS-AHEAD Study, the IQCODE questionnaire for cognitive decline in the elderly [37], and items used in the Pittsburgh cohort studies of aging [38].
  - A score of 5 or less on the Six-Item Screener (SIS) [39]. The SIS is a global measure of cognitive status that assesses three-item recall and orientation to year, month, and day of the week [39]. The SIS screening test was developed in the primary care population at Eskenazi Health and validated in a racially diverse population. Scoring is determined by the number correct with a range of 0–6, where higher scores indicate better cognition [39–41].
5. Able to communicate in English
6. Access to a telephone

After reviewing the initial 6 months of recruitment, the study team recognized that certain eligibility requirements significantly compromised the ability to meet recruitment targets. With approval from the Data and Safety Monitoring Board (DSMB), we subsequently expanded criterion 2 to include those who achieved a calculated measure of anticholinergic medication exposure based on medical record data from the prior 12 months, and removed the requirement for criterion 4a and 4b (presence of subjective cognitive decline defined through either approach). We continued to collect data on the presence of subjective cognitive complaints in a similar manner as outlined above.

Exclusion criteria:

1. Permanent resident of an extended care facility (nursing home)
2. Diagnosis of schizophrenia, bipolar disorder, or schizoaffective disorder defined by ICD9/10 codes
3. Diagnosis of ADRD as determined by either:
  - ICD-9/ICD10 codes
  - Current use of a medication for ADRD (donepezil, rivastigmine, galantamine, or memantine)
  - A pattern of responses to the Functional Activities Questionnaire (FAQ) that suggest a combination of assistance and dependence in daily activities that is consistent with level of function in ADRD (i.e., exclude if ≥ 3 FAQ items are scored at “requires assistance,” or if ≥ 1 FAQ item is scored at “dependent”) [42].

### Randomization procedures

In this cluster randomized trial, randomization occurs at the level of primary care provider in a 1:1 proportion between intervention and usual care using a randomization list with a computer-generated random scheme in random blocks stratified by healthcare system. Research staff are blinded to provider allocation status at the time of recruitment and throughout the study to avoid bias in data collection.

### Deprescribing intervention

The deprescribing intervention is executed through a trained clinical pharmacist employed within each study site, called a deprescribing care coordinator (DCC). This model is developed from prior experiences in anticholinergic deprescribing in our local institutions [33, 43–45]. Training is provided in geriatric care (particularly high-risk medications and appropriate alternatives), educational methods, titration and monitoring schedules, and behavioral economic principles that encourage communication and successful deprescribing attempts. The latter emphasizes the use of messengers, identifying incentives, and setting defaults that can support deprescribing. The trained clinical pharmacists communicate the deprescribing attempt with the patient, providers, and pharmacy as needed to coordinate the transition from a high-risk anticholinergic medication to a safer alternative.

The following general steps are conducted by the DCC to complete the deprescribing process: (1) clarifying the indication for the anticholinergic through medical record review and participant interview; (2) education of potential risks of cognitive impairment in older adults; (3) making a shared decision to deprescribe among participant, caregiver (as needed), and providers; (4) identifying an appropriate alternative (not an anticholinergic medication or potentially inappropriate medication for older adults as defined by the American Geriatrics Society’s Beers Criteria ® [46]) if desired; (5) communicating the deprescribing plan with the appropriate provider(s); and (6) monitoring and revising the plan as needed. The deprescribing process is initiated within 7 days of enrollment, and most deprescribing is expected to be completed within 4 to 6 weeks after enrollment (see appendix).

The participant, pharmacist, and physician will decide which deprescribing approach is most appropriate. Timelines for taper schedules will follow recommendations in the appendix regardless of the intent to replace the anticholinergic with a non-anticholinergic alternative or non-pharmacologic approach. Modification to tapering intervals will be allowed based on clinical judgment of the research or clinical staff (DCC or provider physician) along with monitoring of symptom burden. If multiple anticholinergics are being used concurrently, a drug-specific decision on the deprescribing approach will be necessary, and only one deprescribing attempt will be performed at a time. According to the deprescribing protocol, tapering/discontinuation attempts will follow either a concurrent crossover, delayed crossover, or tapered discontinuation plan (see Appendices 1 and 2 for additional recommendations); however, this process is subject to interruption and delay based on clinical context for each participant and agreement between the DCC and providers caring for the participant.

**Fig. 4:**
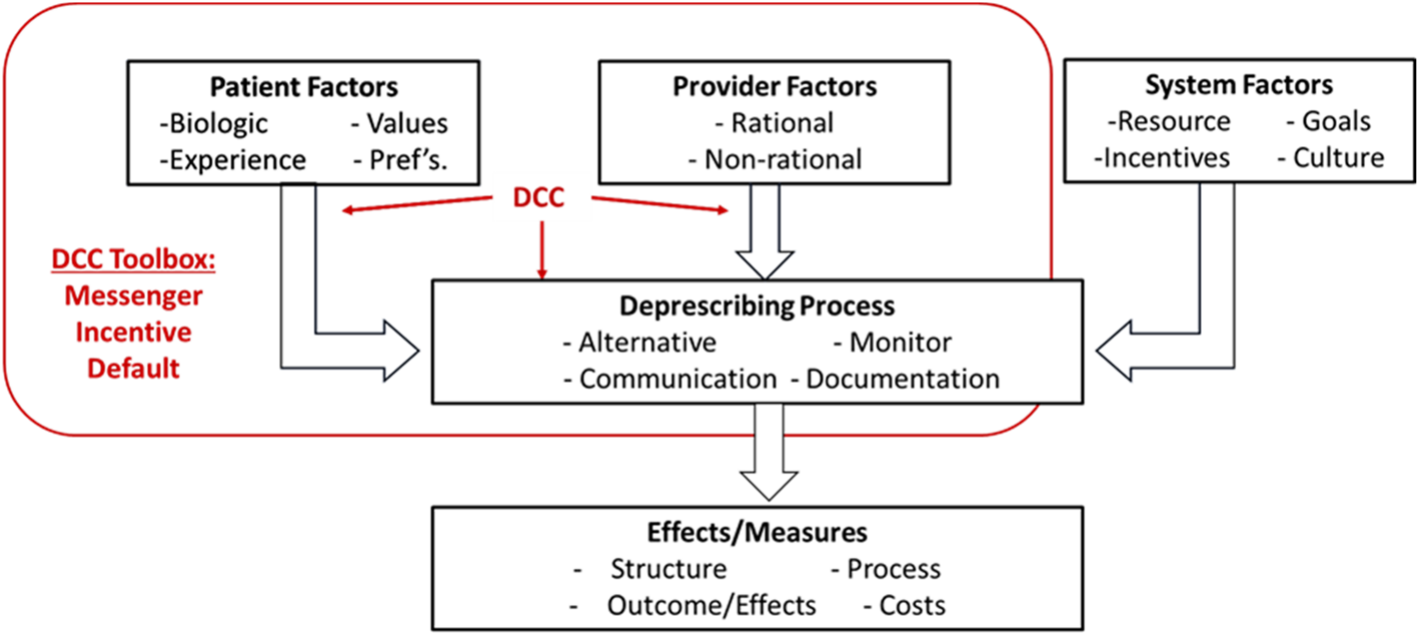
Factors to be influenced by the pharmacist deprescribing care coordinator

The design of the intervention acknowledges a number of factors that influence deprescribing success as recognized in the general model published by Linsky and colleagues (see Fig. 4) [47]. These include patient factors, provider considerations/factors, and the process of deprescribing (medication orders, monitoring plans, and adjustment based on patient-reported outcomes). Furthermore, our model of deprescribing anticholinergics includes the influence of behavioral economics on each of these factors. Recognizing the role of the messenger, incentives, and default settings in the process of deprescribing is addressed in the deprescribing protocol and contributes to the implementation of the intervention.

### Usual care

The group randomized to usual care will receive educational material via traditional mail that reviews risks of polypharmacy, but no information specific to anticholinergic medications. The material will direct participants to their prescribers or pharmacists if they have concerns about their medications. The information will be delivered approximately 7 days following enrollment to coincide with the onset of the intervention in those randomized to the intervention group.

### Measures

The primary outcome measure is cognitive performance in three critical domains relevant to the diagnosis of dementia that include information processing speed, memory, and executive function. In older adults, these domains have been shown to be impaired by anticholinergic medications [48]. We originally selected four cognitive tests based on a systematic review of 78 RCTs among healthy adults that identified tests that were sensitive to the adverse cognitive effects of medications [48]. These included the Digit Symbol Substitution Test (information processing speed), Buschke selective reminding task (memory and new learning), Choice Reaction Time, and Trail Making Test Parts A and B (executive cognitive function) [49–51].

Because approval to initiate recruitment was received only days before in-person research operations were interrupted by the COVID-19 pandemic, we identified an alternative battery of cognitive tests encompassing the same domains but with the ability to be administered remotely with equivalent validity. Therefore, we modified the battery for the primary outcome to include the Symbol Digit Modalities Test (information processing speed), Hopkins Verbal Learning Test (memory and new learning), Oral Trails Making Test (executive function), and Controlled Oral Word Association and Semantic Fluency tests (executive function including phonemic and semantic fluency). These tests have been used by our team with fidelity across telephonic modes of delivery [52].

We will form an overall cognitive composite score as the average of each cognitive measure’s *z*-score, which is constructed by subtracting the mean baseline scores and dividing by the baseline standard deviation. Cognitive measures will be taken at baseline, 6, 12, 18, and 24 months during scheduled outcome assessment visits conducted by telephone. Parallel alternate forms will be used for all tests (3 versions each) presented in a counterbalanced fashion throughout the trial thus minimizing confounding due to learning effect.

The study intervention proposes to reduce exposure to medications that are prescribed to manage symptoms of depression, anxiety, insomnia, and pain. We will use the Patient-Reported Outcomes Measurement Information System (PROMIS) to capture the four patient safety outcomes. PROMIS was developed by the National Institutes of Health. It is unique in its ability to standardize scores across diagnoses and symptoms, it possesses excellent reliability and validity, and it is inclusive of diverse population characteristics and backgrounds [53]. The PROMIS profile-29 (www.nihpromis.org) includes brief scales for several domains; we will use four-item scales for depression, anxiety, and sleep disturbance (insomnia) and a 3-item pain intensity scale [53]. These and other safety measures (see below) will be collected at baseline and months 6, 12, 18, and 24. Comprehensive scoring manuals are available for each measure with guidance for interpreting individual and composite scales. Each PROMIS raw score can be converted to a *T*-score where 50 represents the general population norm for that symptom and each 10-point deviation represents one standard deviation (SD) from the population norm.

We will use the 15-item Health Utility Index (HUI) to determine each participant’s health-related quality of life (HRQOL) at baseline and months 6, 12, 18, and 24 [54]. The HUI is a utility-based HRQOL instrument applied in patients with a wide range of medical conditions. It has eight attributes: vision, hearing, speech, ambulation, dexterity, emotion, cognitive function, and pain. The individual health domain scores range from 0.00 (maximum impairment) to 1.00 (no impairment) and the multi-attribute (HUI) scores, a multiplicative function of individual attribute levels, range from 0.36 to 1.00 with anchors 0.00 = dead and 1.00 = perfect health.

To assess adverse events, two methods are planned: (1) a single-item question will be asked at each follow-up assessment to collect self-reported adverse events, and (2) acute care utilization, defined as emergency room or hospitalization recognized through our statewide health information exchange (Indiana Network for Patient Care). Both methods will be reviewed and summarized at each meeting of the DSMB.

The presence of ApoE Ɛ4 alleles may modify the effect of anticholinergics on cognitive impairment; therefore, we originally planned to collect blood from each participant at baseline to include as a covariate in the responder analysis (secondary aim). However, given the COVID-19 interruption of in-person research, we revised our procedures to collect the APOE Ɛ4 variable remotely through saliva samples. Genomic DNA will be extracted from saliva samples using the DNeasy Blood & Tissue Kit (Qiagen, Inc., Valencia, CA) according to the manufacturer’s protocol. Sample kits will be distributed by mail to participant homes with instructions on how to complete and with pre-paid packaging to return to the Indiana Biobank for storage and processing. The sample kit will be distributed at the beginning of the study, but may be completed at any time of participation, and must be completed before 24 months or withdrawal from the study. Extracted DNA will be sent for genotyping.

Because individual differences have potential to modify treatment outcomes, we will measure the following demographic and clinical factors: age, self-reported race, self-reported gender, self-reported years of education, and relevant clinical diagnoses impacting cognition such as depression, cardiovascular disease, stroke, and diabetes as we have done in prior studies [8, 17, 55]. These demographic variables will be collected at baseline while diagnoses from the electronic medical record will be collected at baseline and 24 months (see Table 1). We will collect medication use through both self-report at baseline and each outcome assessment, and extract prescribed medications (medication orders) from electronic medical records prior to enrollment as well as during the 24-month study period.

**Table 1:** Planned outcome measures and covariates

Variables/outcomes	Baseline	6 months	12 months	18 months	24 months	Method
Demographic characteristics	X					Self-report
Clinical characteristics/diagnoses	X				X	Self-report and EMR
Self-reported medications	X	X	X	X	X	Self-report
Prescribed medications	X				X	EMR
Cognitive performance	X	X	X	X	X	Performance
PROMIS 4-item scales: - Pain - Anxiety - Insomnia - Depression	X	X	X	X	X	Self-report
Health Utility Index	X	X	X	X	X	Self-report
ApoE Ɛ4	X					Saliva

*ApoE Ɛ4* Apolipoprotein Ɛ4*, EMR* Electronic medical record, *PROMIS* Patient Reported Outcome Measurement Information System

### Data management

Study data are collected and managed using the Research Electronic Data Capture (REDCap) electronic data capture tools supported by the Clinical and Translational Sciences Institute at Indiana University [56, 57]. REDCap is a secure, web-based software platform designed to support data capture for research studies, providing (1) an intuitive interface for validated data capture; (2) audit trails for tracking data manipulation and export procedures (auditing is independent of investigators and sponsors); (3) automated export procedures for seamless data downloads to common statistical packages; and (4) procedures for data integration and interoperability with external sources.

### Analysis plan

The cluster randomized trial design will be used by randomizing primary care physicians (PCP) into the intervention or usual care arms. Outcomes will be collected and analyzed at the patient level. We will examine assigned randomization against the computer-generated randomization scheme to ensure randomization validity. Patient characteristics between the two groups will be compared using mixed effects models for continuous variables and generalized estimating equations for categorical variables while adjusting for random PCP effects. We will examine the distributions of continuous variables and use alternative approaches such as transformation or nonparametric methods in cases of violation to the normal distribution assumption. We will also examine the frequency distribution of all categorical variables and adopt exact inference procedures in cases of zero or small cell size. Any baseline variable that is found to be significantly different between the two groups will be included as a covariate in the analyses outlined below.

An intension to treat approach will be used in the analysis of all outcome measures. Composite cognitive scores collected at 6, 12, 18, and 24 months will be used as the primary outcome variables in mixed effects models. An indicator variable for intervention group (intervention vs usual care), time, interaction between group and time, and baseline cognitive scores will be included as independent variables as well as significant factors from the baseline comparison. PCP will be used as random effects to adjust for within-physician correlations. Significant group and time interaction or significant group main effect will indicate differences in cognitive function between the two groups. If significant interactions between group and time are found, post hoc analyses will be conducted to determine the times when the two groups have significantly different cognitive scores. A significant main group effect in the absence of a group-by-time interaction would indicate differences between groups in cognitive function at all follow-up time points.

In a second set of models, we will determine whether differences between groups were accounted for by decreases in total exposure to anticholinergics from baseline to 24 months. For each medication, standardized daily exposure will be calculated using standardized daily dose as in the study by Gray [7] and validated in our prior work [14, 17, 33] in which the daily dose is divided by the minimum effective dose. Days of standardized exposure for each medication will be the sum of the standardized days of exposure in a time period that includes each medication for each patient. This exposure is defined analogously to the pack-years concept used to characterize exposure to cigarette smoking and is a cumulative measure reflecting both exposure intensity and duration. Total exposure will be compared between the two groups using mixed effects models with group (intervention and control) as the independent variable adjusting for significant baseline variables and random PCP effects. We will then use the mixed effect model with cognitive scores at 6, 12, 18, and 24 months as outcome variables described above with total anticholinergic exposure as an additional independent variable adjusting for baseline cognitive scores and PCP random effects. Attenuation of the intervention effect from the original model would indicate that the effect of intervention is mediated through the reduction in anticholinergic exposure. Comparisons of PROMIS scores and HUI will be conducted similarly to the models we described above for cognitive scores. Exploration of potential moderation effects from *ApoE*, or participants’ characteristics will be conducted by including each of these variables and its interaction with the randomization group in the mixed effects models for the outcome measures. Significant interactions will indicate significantly different intervention effects in subgroups defined by *ApoE*, or participants’ characteristics.

Interim analyses will not be conducted to evaluate conditional power (futility) or efficacy, thus preserving the overall type I error probability regarding the efficacy of the intervention. Given the nature of an elderly study population, we expect study attrition in the form of participant death or withdrawal. Our proposed analysis plan using mixed effects models are robust under the missing at random (MAR) assumption that the probability for dropping out depends on participants’ characteristics and medical conditions. We will compare patients’ characteristics between the completers and non-completers to detect potential violation to the missing at random assumption. We will conduct sensitivity analyses to assess the potential impact of missing data using multiple imputation which allows the imputation of missing outcomes for those who drop out using data gathered from these patients at baseline and prior to drop-out. We will use SAS software version 9.4 (SAS Institute, Cary, NC) or relevant updated versions for all analyses.

### Sample size and rationale

The sample size calculation was conducted using a mixed effects model and adjusting for the cluster randomization design. Assuming a within-subject correlation of 0.3, 0.2, and 0.1 for outcomes observed 6, 12, and 18 months apart, respectively, from the same individuals over time, 96 patients per group is required for 80% power to detect differences in composite cognitive scores of 0.6 SD at 24 months between the intervention and usual care groups with type I error rate at *α*= 0.05 assuming linear trends in cognitive change in both groups. We further assume an intra-class coefficient (ICC) of 0.05 for primary care physicians and an average of 6 patients per physician. Accounting for the cluster design requires 120 patients per group to complete the trial. With an expected 30% attrition rate, we plan to enroll 172 patients per group into the study. Prior studies have reported mean differences in 1-year change of cognitive scores of 1.82 (SD 17) or 1.93 (SD 15) between those on definite anticholinergics versus those non-users. The effect sizes we used assume that our intervention will be able to detect a one-sixth reduction in expected cognitive decline if patients in the intervention group discontinue anticholinergics. Our assumption on the ICC is based on prior studies on prescribing patterns in physicians and practices [58].

In primary aim 2, using the effective sample size of 96 patients per group accounting for the design effect from cluster randomization, we will have 85.6% power to estimate the confidence bands with half-width of 0.3 SD for the differences in symptom scores from PROMIS and HUI scores between the intervention and usual care groups during follow-up using the two-sample *t*-tests at the 0.05 level. Power estimates were calculated using the GLMPower and Power procedures in SAS software version 9.4 (SAS Institute, Cary, NC).

### Ethics review and approval

All procedures have received and maintained ethical approval from the Institutional Review Board of the Indiana University (IRB# 1,811,254,189; Federal-Wide Assurance Number, FWA00003544). As a multisite clinical trial, a reliance agreement was established to retain the Indiana University IRB as the single site ethical review board. The trial has also been registered at ClinicalTrials.gov (Identifier: NCT04270474).

The study employs an external DSMB including a senior investigator trained in geriatrics (chair), a biostatistician, a pharmacist/pharmacy services researcher, and funding agency representatives. The DSMB approved the study procedures and plans to meet at 6–9-month intervals or as needed to review safety data.

The study received approval to initiate recruitment in March 2020 just days before a national interruption of research activities as a result of the global pandemic. In response, the study team revised operations to shift to fully remote procedures to avoid further delay in initiating and executing the trial. This included revisions to the process for recruitment, data collection, and measures employed. In June 2020, these changes were approved by the IRB, the DSMB, and the National Institute on Aging (NIA), with recruitment starting in July 2020.

## Discussion

The R2D2 study will be the first to evaluate the impact of deprescribing strong anticholinergic medications on measures of cognition captured up to 2 years following the deprescribing attempt. Key elements of the study design include cluster randomization to minimize risk of contamination and a 2-year follow-up phase to evaluate the impact of deprescribing on long-term cognition. The deprescribing intervention utilizes a pharmacist-based deprescribing care coordinator approach to reduce the use of potentially inappropriate anticholinergic medications in primary care older adults. This study will advance the field by addressing the potential causal relationship between anticholinergic medication use and cognition.

In addition to these key features, we note that our approach to the deprescribing intervention intends to test the hypothesis that anticholinergic medications adversely affect cognitive performance. Therefore, the intervention offers alternative, non-anticholinergic medications as alternatives where preferred by participants in the intervention arm. This is not to be confused with other deprescribing approaches that may attempt to reduce the total number of medications (polypharmacy in general). While the intervention does offer a tapered discontinuation without replacement of an alternative medication if acceptable to the participant, it is possible the net impact on the total number of medications will be nil.

We recognize other prospective anticholinergic deprescribing trials that have been conducted, including studies by Moga and colleagues [30], Ailabouni and colleagues [29], Kersten and colleagues [26], and Gnjidic and colleagues [28]. These studies were important preliminary work that either proved feasibility or guided deprescribing approaches in anticholinergic deprescribing; however, only the study by Moga and colleagues identified the potential for improvement in executive function in a subgroup of participants with a higher level of amyloid-beta pathology. Other studies failed to find differences in cognitive outcomes, though three were preliminary studies limited by sample size, and one was limited by frailty of the study population or duration of follow-up. Our approach is unique in its exclusive focus on strong anticholinergic medications, target population of primary care older adults without dementia, and duration of follow-up. Additionally, our planned sample size of 344 represents the largest randomized trial of anticholinergic deprescribing conducted to date.

Some limitations of the study are worth noting. First, while the cognitive outcomes employed in this study represent domains relevant to the diagnosis of dementia, we recognize that performance on cognitive outcomes alone is only one component of the diagnosis of dementia. If our hypotheses hold, and a differential effect on cognition between groups is identified, future work could extend the intervention and determine the impact on the diagnosis of dementia and time to diagnosis of dementia. Secondly, the study as proposed lacks a measure of the mechanism through which the causal relationship between anticholinergics and cognition are linked. While financial and scientific support for the planned trial is acknowledged, the resources were insufficient to include biomarkers of the mechanism through which anticholinergics may lead to cognitive impairment. We are unable to include biomarkers of anticholinergic use and cognitive impairment to correlate changes in measures of medication use with brain health. Finally, we collect two sources of medication use: self-reported medication use at 6-month intervals and medication prescriptions (orders) from the electronic medical records. These sources introduce advantages and disadvantages, however represent the data sources available to the study team throughout the conduct of the study.

### Trial status

The study began recruitment on July 20, 2020 and is currently recruiting participants. At the time of drafting of the manuscript, approximately 328 participants have been enrolled and is expected to be completed by July 31, 2024. Data collection is expected to be completed by July 31, 2026.

## Supplementary Information

Additional file 1: Appendix 1 Pharmacist-based deprescribing with behavior change influence for R2D2 study protocol.

Additional file 2: Appendix 2 Anticholinergic deprescribing recommendations for R2D2 study protocol.

Additional file 3: SPIRIT checklist.

## Data Availability

Data will be made available upon written request from researchers, and requested data should include information about the identity of the investigator(s), data requested, and plans for data analysis. Trial results will be shared on ClinicalTrials.gov, published in peer-reviewed scientific publications, and presented at state, regional, national, and possibly international scientific meetings.
