# Supplementary material for: Deprescribing anticholinergics to preserve brain health: reducing the risk of dementia through deprescribing (R2D2): study protocol for a randomized clinical trial
Source: Trials. 2024 Nov 22;25:788. doi: 10.1186/s13063-024-08618-4 (PMC11583418; doi:10.1186/s13063-024-08618-4)
Supplement: Supplementary file 1 — Additional file 1: Appendix 1 Pharmacist-based deprescribing with behavior change influence for R2D2 study protocol. [file 13063_2024_8618_MOESM1_ESM.docx]

**1. Purpose**

This protocol details the execution of the pharmacist-delivered intervention to be performed by the study pharmacist within each health system. The recommendations in the following deprescribing protocol are intended to define expected processes for the safe deprescribing of anticholinergic medications to determine the impact on cognition (primary outcome) and the potential reduction in the risk of cognitive impairment. Safety measures are also planned to determine the impact of the intervention on symptom burden.

**2. Referenced Documents**

**2.1. ACB Scale**

**2.2. Anticholinergic class specific taper and monitor recommendations**

**2.3. Intervention documentation/Fidelity measures**

**3. Procedure**

**3.1.1. Introduction to Anticholinergic Deprescribing Recommendations**

Deprescribing is often performed in clinical environments to reduce the number of medications a patient may be using. While multiple definitions of deprescribing exist, more recent versions place a consistent priority on deprescribing for the purpose of reducing unnecessary medications or those that may be causing harm while not providing an expected benefit. This deprescribing intervention is not intended to merely reduce the number of medications, and in fact is not designed to merely reduce the number of medications, but rather reduce the number of high-risk medications, defined here as anticholinergics. As such, reducing or stopping an anticholinergic is the goal of this deprescribing protocol, however replacing with a safer alternative medication is also acceptable, which may have a neutral impact on the overall number of medications. The primary objective of this protocol is to evaluate whether anticholinergic medications are causing harm through adverse cognitive effects, not whether reducing the number of medications influences cognition.

**3.1.2. Behavioral Economics & Choice Architecture Influences**

The design of our intervention intends to accommodate a number of barriers to deprescribing, and incorporates elements from behavior change models to address medication use as a behavior from prescribing (physician) and medication adherence (patient/participant) perspectives. The intervention will also address poor awareness of risk and alternative treatment approaches by providing education to both participants and providers regarding the rationale and potential benefits of deprescribing anticholinergics. Concerns about worsening symptoms or withdrawal events is also a common concern of deprescribing and will be addressed by frequent monitoring by the study pharmacist. Sustainability of a change is also a concern cited by providers after a change is made (represcribing); the surveillance by the intervention pharmacist will minimize the tendency for participants to revert to the use of prior medications.

Behavior-based approaches that may support changing behaviors from physician and participant perspectives may include:

| **Domains of Behavioral Economics** | **Definition** |  | **Patient reported barriers** | **Pharmacist response** |
| --- | --- | --- | --- | --- |
| **Messenger** | Influenced by who communicates | **Highest quality care, this research leading clinical care, agreement from trusted doctor** | “…what would my doctor think of this…?” | ***Your doctor has agreed to participate in this work and supports safe medication changes.*** |
|  |  |  | “…what do you know about medicines…?” "…why should I trust you?..." | ***I’ve been working with these changes in several other people*** |
| **Incentives** | Motivated by loss | **Behavior change to avoid future harm (dementia)** | "…I have been improving on this med…?" | ***This medicine may be good in the short term, but the long term risk may outweigh the benefit. Alternatives are available.*** |
|  |  |  | "…I plan on getting benefit from this med…?" | ***We can provide a medicine that offers a similar effect and that will be safer in the future*** |
| **Norms** | Influenced by what others do | **Describe successful cases** | "…I don’t feel like being bothered with this…" | ***Our experience with X# of patients has shown that this will maximize your benefit and minimize your future risk of harm.*** |
| **Defaults** | ‘go with the flow’ | **Default option is deprescribing** | "…My doctor has been giving me this for years…!" | ***In medicine we are constantly learning new information which is why we are working with your physician to ensure you are taking the safest medications for the future.*** |
|  |  |  | "…I don’t know how to taper this medicine…" | ***We will be walking you through every step of the way so you won’t make a change alone.*** |
| **Salience** | Novel and relevant to us | **Individualize the risk if no change is made** | "…I am worried about the effects of stopping…!" | ***This is a normal concern and we will work with your doctor to provide you a medicine that improves your symptoms now and in the future.*** |
|  |  |  | "…I want a higher dose its not working…" | ***The dose of the current medication may not be the issue, but an alternative medicine may help you reach your long term care goals*** |
|  |  |  | "...I have stopped before and it did not work out…"/"..If I stop I will get symptoms again…" | ***Please describe what happened. We will improve your chances of success by developing a monitoring plan with which you are comfortable*** |
| **Priming** | Influenced by sub-conscious cues |  | "..I am fearful that I won’t be able to do this…?" | ***As your "Brain Health Pharmacist" we will monitor and work with you step by step to ensure safety through this process*** |
| **Affect** | Emotional associations shape actions | **Educate on benefits that provoke an emotional response (identify loss if no change is made)** | "…Do not feel this will work for me…?" | ***I understand the hesitation, but our decisions are based on reducing the future risk of harm.*** |
| **Commitments** | Consistent with public promises | **Share plan and progress with others** | "…I don’t want to disappoint my family…!" "…I am worried about relapse…" | ***This medicine may affect your future brain health so addressing this now may help preserve your quality of life in the future*** |
| **Ego** | Actions that make us feel better about ourselves | **Improve future quality of life** | "…I am taking action against my disease…!" | ***With our help we can improve your medication selection now and reduce future risk.*** |

**3.2 Notification of Patient enrolled in intervention arm**

An email notifying the study pharmacist will be automatically generated by REDCap at the time enrollment data is entered. Emails will be distributed by REDCap according to study site and by randomization status. Note that study pharmacists WILL NOT receive a notification of enrollment of participants in the usual care arm of the trial. The notification email will contain necessary information for the pharmacist to review the participant’s medical record and initiate procedures. This information will include the participant’s Study ID, Medical Record Number, Medications, and Physician Name.

**3.3 Planned Study Contacts & Communication**

The figure below describes the flow of study contacts particularly relating to participants, with planned intervention contacts above the horizontal bar and planned outcome measures (to be conducted by study staff, not the study pharmacist) below the horizontal bar. Following notification of enrollment into the intervention group (see section 3.2 above) the study pharmacist will have 7 days until the first communication is required. This will allow the pharmacist time to review the medical record and communicate with the provider regarding an acceptable alternative. This will also allow for simultaneous onset of the intervention as the usual care group will receive a general newsletter regarding safe medication practices which will be sent by the study team with an arrival date of roughly 7 days after enrollment.

**
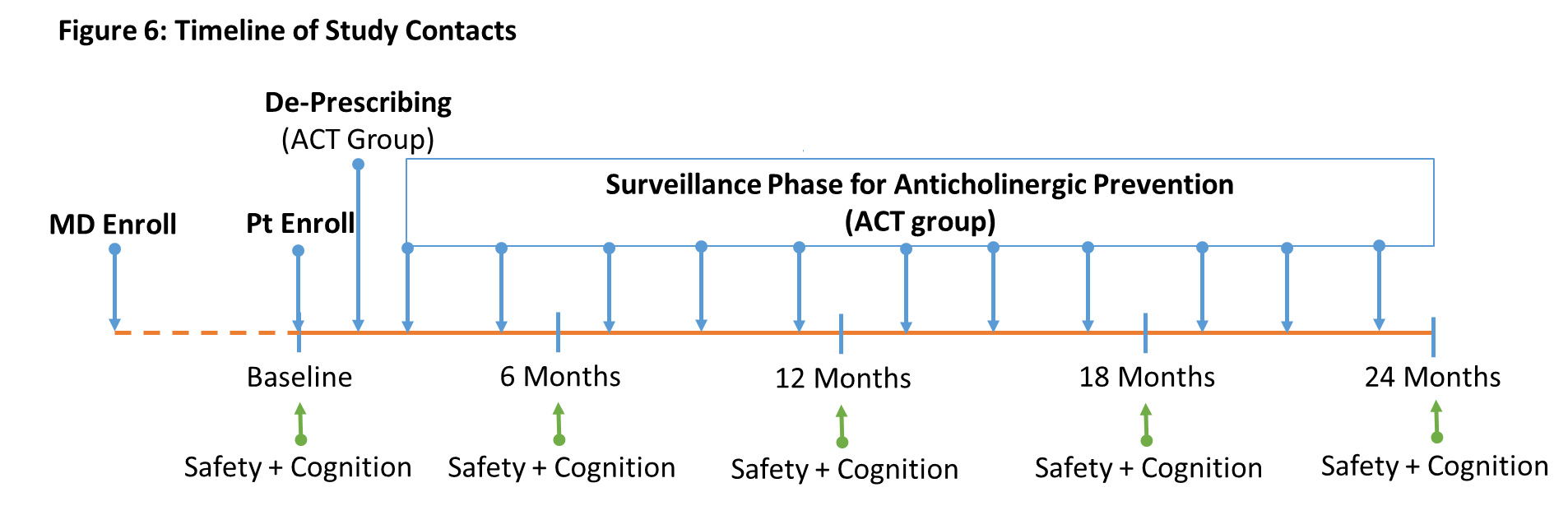
**

**Summary of Planned Study Contacts with Participant:**

- **Baseline/Initial:** Introductory (by phone or face-to-face as requested by patient) to review history and establish plan/timeline for deprescribing. See section 3.3.2. below for details related to participant communication.
- **Deprescribing phase:** Weekly contact by phone or email through deprescribing period (likely 4-8 weeks on average). See appendix 2.2. for general deprescribing recommendations.
- **Surveillance Phase:** Monthly review of medication orders from Regenstrief data reporting (Epic/Cerner) among active participants (individual participant contact not required)
- **Surveillance Phase:** Bi-monthly (every other month) contact from intervention team by phone or email.

**3.3.1. Physician Communication**

The intervention pharmacist will serve as the central source of communication between participant, providers, and (as needed) dispensing pharmacy to coordinate the de-prescribing process. Following the discussion of participant preferences, the pharmacist will communicate with the primary care provider to define the most appropriate alternative medication, titration schedule, and monitoring plan. In cases where a specialist is the prescriber of the target anticholinergic, the pharmacist will communicate with the specialist and communicate the plan with the primary care physician throughout the duration of the study period (24 months for each participant). Messages to physicians should be sent through the mailing system through the relevant electronic medical record system (Epic or Cerner).

**Initial physician communication should follow the language below:**

*Message subject: R2D2 Study Plan*

“Your patient, ***­­­­______________***, has enrolled in the R2D2 study, which intends to reduce the risk of harm from high-risk anticholinergics. Per the study protocol (IRB # ___________), I will be working with her to reduce or stop ***______(insert drug & dose)________*** *they take* ***____(insert pt.-reported frequency)____________.*** Mr./Mrs. __________ will be given the option for a taper to no pharmacologic treatment or a cross-taper directly to a different treatment. As a general rule, the study protocol calls for slow tapers in medications so as not to induce withdrawal reactions or severe recurrence of symptoms.

Upon my review of Mr.Mrs. __________ medical history and concomitant medications, I believe ***_______insert drug, dose, and frequency)_____________________*** would be an appropriate alternative agent if desired. If you have a preference for an alternative treatment or prior experience attempting to deprescribe this medication, please let me know.

**Please respond to this message with approval, objection or alternative suggestions.** ***As the study pharmacist I am willing to write the orders for this change and will communicate Mr./Mrs. _____________ response.*** *I will be communicating with Mr./Mrs. _________ following your response.*

I will follow up with you by phone if no written response is received.”

**Subsequent physician communication during titration period should be as follows:**

*“Per the R2D2 Study Protocol, a change in this patient’s _________ has been made. Mr./Mrs. reports (an increase/ a decrease/ no change) in symptoms as a result of this change.*

*Original Medication Name and Dose:*

*Current Medication Name and Dose:*

*Plan for future change:*

*Planned Alternative (if needed):*

*As always, feel free to message or call me with concerns or other relevant updates.*

*_______________, PharmD”*

**Physician communication for the completion of the deprescribing intervention, entry into surveillance phase:**

“*Per the R2D2 Study Protocol, this patient has completed the attempted deprescribing of ___________. Mr./Mrs . reports (an increase/ a decrease/ no change) in symptoms as a result of this change.*

*Original Medication Name and Dose:*

*Current Medication Name and Dose:*

*Mr./Mrs._____________ will now enter the surveillance phase of the intervention, with a goal of preventing future use of anticholinergics during the remainder of the 24-month total study period. I will be reviewing medication orders and communicating with the patient intermittently throughout this phase to monitor for prescription and over-the-counter anticholinergics, and will re-activate the deprescribing intervention if an anticholinergic medication is initiated.*

*As always, feel free to message or call me with concerns or other relevant updates.*

*_______________, PharmD”*

**3.3.2. Participant Communication**

The study pharmacist is encouraged to send the initial entry communication to the participant’s physician **at least 48 hours before communicating with the participant**. The purpose is to allow the physician time to receive the message and respond with any concerns or suggestions for alternatives or taper schedules.

Mode of communication with the participant will most likely exist through telephone conversations, but as appropriate may be conducted in the participant’s home, by email, or at a mutually agreed upon site such as a meeting room in the participant’s clinic or Regenstrief Institute, whichever allows for private communication and can reliably support the necessary communication. Suggested language for the initial phone call is as follows:

*“Hi Mr./Mrs._______________, my name is ______________________ and I am calling from the R2D2 study in which you enrolled last week. Do you have time to discuss the study and your medications now?”*

(If yes, proceed; if no, reschedule.)

The subsequent conversation should follow the general approach:

*“…I’ve reviewed your medical record, but I’d like to know from you what you are using __________ (insert ACB medication) to treat?”*

*“Tell me a little about how this medication works for you.”*

*“It looks like you’ve tried _________ in the past, how did that work for you?” or “Have you tried anything else for this symptom in the past?”*

*“Did you know that ___________ (insert ACB medication) has some risks, including the possibility of worsening your memory and increasing your risk of dementia?”*

*“Did you know that some other medications also treat __________ (symptom) and are not likely to increase your risk of dementia. This trial would like to find out if you are receiving a benefit from this medication in treating ­________ (symptom) and if you need a different medicine for this symptom, how you well you might do on the other medication and see if your brain is healthier as a result of using safer medicines.”*

*“I will work with your doctor and help you slowly decrease your dose so that your symptoms don’t get worse, and decide if an alternative medication would work for you and keep your brain safer as you age.”*

**3.4 General Deprescribing Recommendations, Selection of alternatives, tapering, and monitoring**

Decision constructs including patient, medication, and disease-specific factors are described in the figure, which prioritize patient safety through integrated and personalized care. The goal of the protocol is to incorporate medication-specific factors from the Scott and Linsky frameworks for minimizing inappropriate medication use and process of deprescribing. We also emphasize patient preferences (efficacy, adverse effects, and cost) as a key component informing the deprescribing process. Lastly,
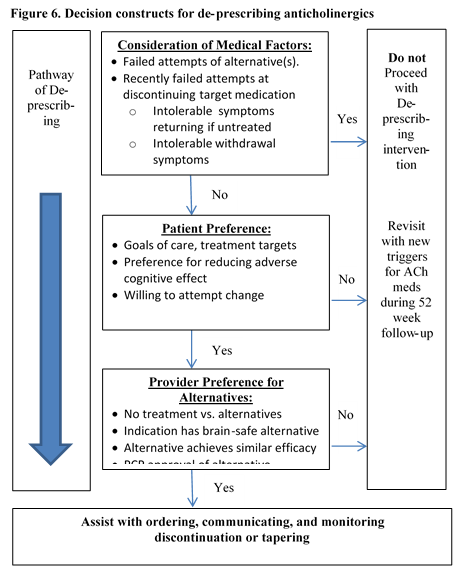
providers will be integrated into the selection of alternatives and monitoring process. Aligning each of these components optimizes the likelihood of successful changes in therapy.

**The goal of the deprescribing intervention is an absolute reduction in anticholinergic burden from strong anticholinergics; prior literature suggests that cognitive improvement is likely only experienced among those completely discontinuing all anticholinergics** (Kersten, et al. 2013).

A summary of the steps to making changes follows:

Step 1: Pharmacist reviews medical record, identifies key medical history, concomitant medications, prior attempts with non-anticholinergic alternatives

Step 2: pharmacist selects non-anticholinergic alternative and appropriate taper schedule and timeline

Step 3: Pharmacist communicates plan and intent with appropriate physicians (primary care and, if necessary, specialist or ordering physicians), making adjustments as requested and clinically appropriate

Step 4: Pharmacist communicates plan with participant, incorporating any new information into the plan and communicating with physicians as needed.

Step 5: pharmacist makes arrangements to execute plan, writing medication orders and communicating with pharmacy as needed (cancelling/discontinuing anticholinergic as appropriate).

Step 6: monitor and communicate execution of deprescribing plan as designed and with attention to modifications as needed

**3.4.2. Selection of Alternatives**

The intervention pharmacist will review medical record data to identify comorbidities, concomitant medications, reason for anticholinergic use, and whether alternatives or deprescribing trials have been previously attempted. Based on chart review, the pharmacist will identify potential alternatives, paying attention to potential drug interactions and appropriateness of alternatives. The pharmacist will be expected to suggest an appropriate alternative and taper schedule and provide the justification to the primary care (and if appropriate specialist) physician. Upon approval or assent, the pharmacist will educate the patient regarding the rationale for the deprescribing recommendation and discuss the deprescribing approach (cross taper, delayed taper, or tapered discontinuation to non-pharmacologic management). At this time, the pharmacist will also elicit patient preferences that influence alternative selection, such as adherence, side effects, and cost, and adjust the plan as needed while also communicating any changes with physicians.

Our list of alternative pharmacologic and non-pharmacologic therapies have been used in our prior work (See appendix). This list of alternatives has been informed by recommendations from the American Geriatrics Society. Alternatives suggested by the intervention pharmacist will account for participant, indication, and disease-specific characteristics. Participants are not restricted to recommended alternatives, but will be directed towards medications devoid of anticholinergic effects or other adverse cognitive effects (including benzodiazepines).

**3.4.3. General tapering recommendations for deprescribing**

The patient/participant, pharmacist and physician will decide which of the following approaches to deprescribing is most appropriate for the patient/participant. Timelines for taper schedules will follow recommendations in the appendix below regardless of the intent to replace the anticholinergic with a non-anticholinergic alternative or non-pharmacologic approach. However, modified intervals for tapering will be allowed pursuant to the clinical judgment of the research or clinical staff (research/intervention pharmacist or intervention physician). If multiple anticholinergics are being used concurrently, a drug-specific decision on the deprescribing approach will be necessary, and only one medication change should be performed at a time.

3.4.3.A. Concurrent Crossover

Cross-taper from ACB medication directly to non-anticholinergic alternative.

3.4.3.B. Delayed Crossover

Taper down ACB with goal of discontinuation, monitor symptoms without treatment, then determine if non-anticholinergic alternative is desired.

3.4.3.C. Taper to non-pharmacologic treatment

Taper ACB medication down with goal of discontinuation while educating and encouraging adherence to non-pharmacologic treatments alone (such as sleep hygiene or bladder care).

**3.4.4. Monitoring**

Pharmacists will monitor for any adverse events and document those adverse events in the medical record and study database. Adverse events may include *adverse drug reactions* as well as *adverse drug withdrawal events*. Adverse drug withdrawal reactions may occur as a physiologic reaction to removing the original medication. Adverse drug reactions may occur as a reaction to a new/alternative medication. The pharmacist should use clinical judgment and evaluate for both occurrences.

Additionally, the study pharmacist will monitor participant-reported symptom burden before, during, and after a deprescribing trial. The participant-reported symptom burden will guide deprescribing tapers and will be based on symptom burden reported at baseline on the existing regimen. After each dose change/taper, symptom burden should be assessed. If the participant reports no change or even improvement in symptoms relative to baseline, the deprescribing attempt should proceed as planned. If symptoms worsen, clinical judgment and participant-preference should be considered in the plan to proceed with deprescribing. In these cases, rescue plans may include resorting to the last dose tolerated, extending the taper duration, or increasing the dose of an alternative medication may be considered.

**3.4.5. Managing medication changes within collaborative practice agreement**

Orders for prescription medication changes among participants in the intervention group may be composed and executed by pharmacists pursuant to the terms of an approved collaborative practice agreement with physicians enrolled in the trial and randomized to the intervention arm. Orders may include dose titrations, new orders, and discontinuations of both anticholinergic medications (for the purposes of reducing the dose and discontinuing) as well as non-anticholinergic alternative medications.

**3.4.6. Managing medication changes through verbal orders**

Pharmacists executing the study intervention outside of an approved collaborative practice agreement will make changes in prescription medications (both anticholinergics and non-anticholinergic alternatives) only with written or verbal approval from the primary care or ordering physician.

**4.0 Documentation**

**4.1. REDCap**

REDCap documentation will be required to capture participant communication/contacts and remind the study pharmacists of timeline for contacts in the surveillance phase.

**4.2. Deprescribing measurement and Fidelity Data Capture**

The Excel file “R2D2 Fidelity Measures” will be used to capture participant-specific measures of the intervention. These measures will include:

- Baseline ACB drug, dose, & estimated annual TSDD
- Deprescribing approach: concurrent crossover, delayed crossover, or taper to non-pharmacologic management
- Duration of taper (# of weeks)
- Emergent adverse events (yes/no and comment field)
- Emergent withdrawal events (yes/no and comment field)
- Outcome of deprescribing baseline ACB: (no change, lower dose, discontinuation, restart) – to be completed immediately after taper and every 6 months until the end of trial participation.

CONTACT RESEARCH COORDINATOR OR PRINCIPAL INVESTIGATOR WITH ANY QUESTIONS OR CONCERNS.
