# Supplementary material for: Deprescribing anticholinergics to preserve brain health: reducing the risk of dementia through deprescribing (R2D2): study protocol for a randomized clinical trial
Source: Trials. 2024 Nov 22;25:788. doi: 10.1186/s13063-024-08618-4 (PMC11583418; doi:10.1186/s13063-024-08618-4)
Supplement: Supplementary file 2 — Additional file 2: Appendix 2 Anticholinergic deprescribing recommendations for R2D2 study protocol. [file 13063_2024_8618_MOESM2_ESM.docx]

**Class-Specific Deprescribing Recommendations for Strong Anticholinergics**

*Adapted from the intervention protocol supporting the “Reducing the Risk of Dementia through Deprescribing (R2D2)” cluster-randomized trial conducted by Purdue University, Indiana University, and the Regenstrief Institute and supported by the National Institute on Aging (R01AG061452), and developed with the Eskenazi Health - Healthy Aging Brain Program. These recommendations represent expert opinion rooted in evidence where it exists.*

| **Proceed with caution**  **With any of below diagnoses** | **Titration Plan** | **Recommended Alternatives** | **Monitoring Recommendation** | **Restart Characteristics** |
| --- | --- | --- | --- | --- |
| **Anticholinergic Antipsychotics**  Olanzapine, Quetiapine, Clozapine, Chlorpromazine, Thioridazine, Perphenazine, Trifluoperazine | | | | |
| Dose reduction before discontinue or change if the following:  Schizophrenia, Schizoaffective disorder, Bipolar disorder  Tourette's syndrome, Tic disorders, autism  Mental retardation, autism, developmental delay  OCD, alcoholism, cocaine abuse  (exclusion criteria for R2D2 study) | If use is longer than 6 weeks, taper off over at least 2 weeks (low dose) or 4-8 weeks (medium/high dose). Dose titrations suggested at 2 week intervals. | Insomnia:   - Sleep hygiene - Melatonin - Trazodone   BPSD:   - PREVENT non-pharmacologic interventions   Depression:  (Select alternative with psychiatrist if provider)   - Aripiprazole - Combination antidepressants | Sleep hygiene, sleep duration, daytime wakefulness and activity weekly until discontinued  Behavioral symptom monitoring at weekly intervals until 1 week after last titration or discontinuation.  ADWE: agitation, fear or hallucinations, nausea, vomiting, increased sweating or sleeping problems. | Recurrence of severe symptom/insomnia upon withdrawal, non-pharmacologic options failed, and participant is a threat to self or others |
| **References:**  1. Declercq T, Petrovic M, Azermai M, SR. V, De Sutter AI, van Driel ML, et al. Withdrawal versus continuation of chronic antipsychotic drugs for behavioural and psychological symptoms in older people with dementia. Cochrane Database Syst Rev 2013;3:Cd007726.  2. Rosenheck RA, Leslie DL, Sindelar JL, Miller EA, Tariot PN, Dagerman KS, et al. Cost-benefit analysis of second-generation antipsychotics and placebo in a randomized trial of the treatment of psychosis and aggression in Alzheimer disease. Arch Gen Psychiatry. 2007 Nov;64(11):1259-68.  3. Schneider LS, Tariot PN, Dagerman KS, Davis SM, Hsiao JK, Ismail MS, et al. Effectiveness of atypical antipsychotic drugs in patients with Alzheimer's disease. New England Journal of Medicine. 2006;355(15):1525-38.  4. Sultzer DL, Davis SM, Tariot PN, Dagerman KS, Lebowitz BD, Lyketsos CG, et al. Clinical symptom responses to atypical antipsychotic medications in Alzheimer’s disease: phase 1 outcomes from the CATIE-AD effectiveness trial. The American journal of psychiatry 2008;165(7):844-54.  5. Luchins DJ, Freed WJ, Wyatt RJ. The role of cholinergic supersensitivity in the medical symptoms associated with withdrawal of antipsychotic drugs. Am J Psychiatry. 1980; 137(11):1395–8.  6. Cerovecki A, Musil R, Klimke A, Seemuller F, Haen E, Schennach R, Kuhn KU, Volz HP, Riedel M: Withdrawal symptoms and rebound syndromes associated with switching and discontinuing atypical antipsychotics. CNS Drugs 2013;27:545-572 | | | | |

| **Caution** | **Titration Plan** | **Recommended Alternatives** | **Monitoring Recommendation** | **Restart Characteristics** |
| --- | --- | --- | --- | --- |
| **Anticholinergic Antidepressants**  Amitriptyline, Doxepin, Imipramine, Nortriptyline, Paroxetine | | | | |
| Dose reduction before discontinue or change if the following:  Severe depressive symptoms with objective evidence of failed deprescribing in past 12 months (severe symptom recurrence, suicidal ideation, hospitalization or inpatient psychiatry visit). | If use is longer than 6 weeks, taper off over at least 4 weeks (low dose) or 4-8 weeks (medium/high dose).  If withdrawal occurs, longer taper schedules up to 8-16 weeks may be necessary | Depression:   - Sertraline - Citalopram - Other non-anticholinergic   Anxiety:   - Buspirone - Mirtazapine   Neuropathic pain:   - Duloxetine - Gabapentin - Levetiracetam   Insomnia:   - Sleep hygiene - Melatonin - Trazodone | General mood and withdrawal effects at weekly intervals; suicidal ideation at weekly intervals. Depressive and anxiety symptom scales at two-week intervals as requested by collaborating provider.  Sleep hygiene, sleep duration, daytime wakefulness and activity weekly until discontinued  ADWE: TCA: Lethargy, headache, tremor, sweating, symptoms anorexia, insomnia, nausea, vomiting, diarrhea. Irritability, anxiety/agitation, low mood, symptoms excessive dreaming, nightmares.  ADWE: SSRI: Lightheadedness/dizziness, vertigo, ataxia, numbness, electric shock-like sensations, Lethargy, headache, tremor, sweating, anorexia, Sleep disturbance, Insomnia, nightmares, excessive dreaming , Nausea, vomiting, diarrhea , Irritability, anxiety/agitation, low mood | Recurrence of severe symptom/insomnia upon withdrawal, non-pharmacologic options failed, and participant is a threat to self or others |
| General Recommendations:  1. There is less evidence for continuing antidepressant treatment in older people for a period > 12 months.  **2.** Rapid discontinuation may result in antidepressant discontinuation syndrome. This syndrome is associated with symptoms such as **F**lu-like symptoms, **I**nsomnia, **N**ausea, **I**mbalance, **S**ensory disturbances and **H**yperarousal (anxiety/agitation) (i.e: FINISH). These usually appear one week after abrupt discontinuation of the antidepressant. In patients with more severe withdrawal symptoms, the pre-reduction dose may need to be restarted which results in resolution of symptoms within 24 hours. Tapering can then be reinstated at a slower rate.  **3.** Patients should be monitored carefully as a high risk of suicide attempts exists during dosage changes and discontinuation**.**  **References:**  1. Calati R, Signorelli MS, Balestri M, Marsano A, De Ronchi D, Aguglia E, et al. Antidepressants in elderly: Meta-regression of double-blind, randomized clinical  trials. Journal of affective disorders. 2013;147(1):1-8.  2. Wilkinson P, Izmeth Z. Continuation and maintenance treatments for depression in older people. The Cochrane Library. 2012.  3. Best Practice Advocacy Centre New Zealand (BPAC). A practical guide to stopping medicines in older people, 2010 [cited 2014 20th of May]. Available from: <http://www.bpac.org.nz/BPJ/2010/April/stopguide.aspx>.  4. Arroll B, Macgillivray S, Ogston S, Reid I, Sullivan F, Williams B, et al. Efficacy and tolerability of tricyclic antidepressants and SSRIs compared with placebo for treatment of depression in primary care: a meta-analysis. Ann Fam Med 2005 Sep-Oct;3(5):449-56.  5. Valuck RJ, Orton HD, Libby AM. Antidepressant discontinuation and risk of suicide attempt: a retrospective, nested case-control study. J Clin Psychiatry. 2009;70(8):1069-77.  6. Rosenbaum JF, Fava M, Hoog SL, et al. Selective serotonin reuptake inhibitor discontinuation syndrome: a randomized clinical trial. Biol Psychiatry. 1998;44:77–87. | | | | |

| **Caution** | **Titration Plan** | **Recommended Alternatives** | **Monitoring Recommendation** | **Restart Characteristics** |
| --- | --- | --- | --- | --- |
| **Anticholinergic Bladder Antispasmodic**  Oxybutynin, Tolterodine, Trospium, Fesoterodine, Solifenacin, Darifenacin | | | | |
| Dose reduction before discontinue or change if the following:  Severe pain related to detrusor overactivity in neurological conditions (such as spina bifida). | Reductions in dose may occur as quickly as every 3 days, however weekly changes may be more appropriate and less likely to provoke rebound symptoms. Rebound has been seen with rapid discontinuation.  Alternatives likely require 4 weeks to optimize efficacy; tapers should account for time to onset of alternative | Overactive bladder:   - Scheduled toileting - Pelvic floor exercise - Mirabegron | Incontinence episodes, pain associated with bladder spasm weekly until discontinued or change in therapy.  ADWE:  sudden urge to urinate, involuntary loss of urine, nocturia. | Recurrence of severe pain caused by bladder spasm; interruptive incontinence episodes |
| **References:**  1. Kim A, Lee KS, Kim TB, Kim HJ, et al. Incidence and risk factors of recurrence of overactive bladder symptoms after discontinuation of successful medical treatment. Investig Clin Urol. 2017;58(1):42-47.  2. Lee YS, Choo MS, Lee JY, Oh SJ, Lee KS. Symptom change after discontinuation of successful antimuscarinic treatment in patients with overactive bladder symptoms: a randomised, multicentre trial. Int J Clin Pract. 2011 Sep;65(9):997-1004.  3. Kalder M, Pantazis K, Dinas K, Albert US, Heilmaier C, Kostev K. Discontinuation of treatment using anticholinergic medications in patients with urinary incontinence. Obstet Gynecol. 2014 Oct;124(4):794-800.  4. Gopal M, Haynes K, Bellamy SL, Arya LA. Discontinuation rates of anticholinergic medications used for the treatment of lower urinary tract symptoms. Obstet Gynecol. 2008 Dec;112(6):1311-8. | | | | |

| **Caution** | **Titration Plan** | **Recommended Alternatives** | **Monitoring Recommendation** | **Restart Characteristics** |
| --- | --- | --- | --- | --- |
| **Anticholinergic Central Antihistamines**  Meclizine, Hydroxyzine, Promethazine, brompheniramine, chlorpheniramine, diphenhydramine, clemastine, dimenhydrinate | | | | |
| Dose reduction before discontinue or change if the following:  Recurrence of severe symptoms or adverse drug withdrawal events with de-prescribing trial conducted in past 6 months. | Discontinuation and tapering can be completed from one day to the next for indications of rhinitis, and itching.  Use for nausea or insomnia should be titrated at weekly intervals or every 2 weeks to avoid withdrawal reactions. | Allergic rhinitis:   - Fexofenadine - Loratadine   Itching:   - Moisturizing lotion - Fexofenadine   Insomnia:   - Sleep hygiene - Melatonin - Trazodone   Nausea/vomiting:   - Ondansetron - Procholorperazine | Symptom severity at weekly intervals  Sleep hygiene, sleep duration, daytime wakefulness and activity weekly until discontinued  Nausea, emesis at weekly intervals  ADWE: runny nose, itching, insomnia and irritability. | Recurrence of severe symptoms interrupting daily activities or worsening quality of life |
| **References:**  1. Best Practice Advocacy Centre New Zealand (BPAC). A practical guide to stopping medicines in older people, 2010. Available from: <http://www.bpac.org.nz/BPJ/2010/April/stopguide.aspx>.  2. Hanlon JT, Semla TP, Schmader KE. Alternative Medications for Medications in the Use of High-Risk Medications in the Elderly and Potentially Harmful Drug-Disease Interactions in the Elderly Quality Measures. J Am Geriatr Soc. 2015 Dec;63(12):e8-e18  3. American Geriatrics Society 2015 Beers Criteria Update Expert Panel. American Geriatrics Society 2015 Updated Beers Criteria for Potentially Inappropriate Medication Use in Older Adults. J Am Geriatr Soc. 2015 Nov;63(11):2227-46. | | | | |

| **Caution** | **Titration Plan** | **Recommended Alternatives** | **Monitoring Recommendation** | **Restart Characteristics** |
| --- | --- | --- | --- | --- |
| **Anticholinergic Smooth Muscle Relaxants**  Cyclobenzaprine, methocarbamol | | | | |
| Dose reduction before discontinue or change if the following:  Recurrence of severe symptoms or adverse drug withdrawal events with de-prescribing trial conducted in past 6 months. | Dose reductions should occur at weekly intervals to avoid withdrawal reactions. Longer titration schedules may be necessary with use > 6 weeks. Other titration schedules as instructed by provider. | Muscle spasm:   - Stretching, hydration, therapy as recommended in current pain management plan - Pain management (in collaboration with physicians managing pain syndromes) | Symptom severity at weekly intervals  ADWE: headaches, body aches, irritability, some nausea, anxiety, fever, chills, insomnia, and general feelings of malaise. | Recurrence of pain symptoms interrupting daily activities or worsening quality of life |
| **References:**  1. Hanlon JT, Semla TP, Schmader KE. Alternative Medications for Medications in the Use of High-Risk Medications in the Elderly and Potentially Harmful Drug-Disease Interactions in the Elderly Quality Measures. J Am Geriatr Soc. 2015 Dec;63(12):e8-e18  2. American Geriatrics Society 2015 Beers Criteria Update Expert Panel. American Geriatrics Society 2015 Updated Beers Criteria for Potentially Inappropriate Medication Use in Older Adults. J Am Geriatr Soc. 2015 Nov;63(11):2227-46. | | | | |
